# The Treatment of Pleurisy and Empyema

**Published:** 1893-08-05

**Authors:** 


### PADDINGTON GREEN CHILDREN'S HOSPITAL.

TREATMENT OF PLEURISY AND EMPYEMA—*continued.*

III. *Secondary Pleurisy.*—So far we have been speaking of primary pleurisy, with which no constitutional disease was apparently associated. In all cases a careful examination should be made with the view of detecting some cause for the pleurisy, such as tuberculosis or rheumatism, and regulating the treatment accordingly.

If it be concluded from the physical signs that tubercular disease is present in the lung on the affected side, the cure of a dry pleurisy will probably be much delayed unless constitutional remedies are adopted, and the patient is placed in the best hygienic surroundings. A change to the seaside will do more good than rest in a hospital. If effusion is present, it is found that nourishing diet with codliver oil is more beneficial than early aspiration. The withdrawal of the fluid is frequently followed by a rapid re-accumulation, so that the operation has to be repeated again and again. This is usually accompanied by an extension of the intrapulmonary disease. On the other hand, under rest, counter-irritation, and constitutional treatment, the effusion, as a rule, does not reach an excessive amount, while the signs of pulmonary disease become less marked or disappear.

If the rheumatic diathesis be present, the employment of salicylate of soda gives good results in some cases both of dry pleurisy and of pleurisy with effusion. Aspiration should not be performed, unless the indications are urgent, until a fair trial has been made of anti-rheumatic medicines.

IV. *Empyema.*—When a case of pleural effusion is admitted into the hospital, a hypodermic needle is usually inserted into the chest to ascertain whether pus is present, as empyema is very common amongst children, and the treatment is conducted on quite different lines when suppuration has occurred. In some cases of purulent effusion a preliminary aspiration is made with Potain's apparatus, and if further accumulation takes place the pleura is opened and drained. The aspiration treatment is unsatisfactory, because, from curdiness of the pus, blocking of the tube frequently occurs, and a complete cure seldom follows. The ordinary treatment of empyema is by resection of a rib, and thorough drainage of the pleural cavity.

The patient is anæsthetised, and the skin of the affected side is thoroughly cleansed and disinfected. The spot selected for opening the chest is usually in front of or behind the posterior axillary line, at the level of the seventh rib; but it is of more importance to ascertain by percussion where there is undoubted evidence of fluid, and make the opening over that part. An incision is made in the line of the rib through the skin, subcutaneous tissues, and periosteum down to the bone. The periosteum is stripped off longitudinally until an inch of the rib is exposed. The piece of bone thus bared is separated with bone forceps and removed. The knife is then pushed into the pleural cavity, and a free opening made. In most cases irrigation is employed, with the view of softening and removing any solid masses of pus which may be present. The fluid employed is boiled water, or warm boracic lotion. This is allowed to run into the pleural cavity from a glass vessel raised slightly above the level of the patient. Care is taken that no pressure of fluid occurs inside the chest, and with this object the fluid must run in a gentle stream, and the pleural opening must be large enough to contain the nozzle of the irrigator and at the same time allow of the free exit of the fluid. A certain amount of coughing is useful at this stage, as under its influence the lung will quickly expand up to the opening. If the lung does not expand there is probably some pleural or pulmonary disease which will delay the cure. Should the cough become violent and persistent it is advisable to cease further irrigation, and to dress the wound as quickly as possible. A rubber drainage tube from three to four inches in length, and sufficiently wide to permit the free exit of pus, is introduced, and the wound is dressed with cyanide gauze and salicylic wool.

The after treatment is very important. During the first twenty-four hours the discharge will probably be free, and the dressing will have to be changed once or twice. The discharge then diminishes, and the wound will require to be dressed every second or third day.

Care must be taken that the tube does not get blocked, and after the first day it is shortened at each dressing. Towards the end of the first week, in ordinary cases, the discharge is small in amount and thin and sero-purulent in character. The tube is therefore removed, and the wound in the chest wall is allowed to close up as rapidly as possible. By this means the irritation caused by the presence of the tube in the pleural cavity is checked, and what secretion there is finds its way out at first through the opening in the chest wall, and when that is closed through absorption by the pleural surfaces. The operation wound is usually healed within a fortnight, and the patient is then allowed out of bed. As an attack of empyema is accompanied by considerable wasting in children, nourishing foods with tonics, such as cod liver oil and iron, must be administered for some weeks after the pleural trouble has ceased.

